# The Increasing Relevance of Tumour Histology in Determining Oncological Outcomes in Colorectal Cancer

**DOI:** 10.1007/s11888-015-0280-7

**Published:** 2015-08-02

**Authors:** Iris D. Nagtegaal, Niek Hugen

**Affiliations:** Department of Pathology, Radboudumc, PO Box 9101, 6500 HB Nijmegen, The Netherlands; Department of Surgery, Radboudumc, PO Box 9101, 6500 HB Nijmegen, The Netherlands

**Keywords:** Colorectal cancer, Adenocarcinoma, Mucinous carcinoma, Signet ring cell carcinoma, Neuroendocrine carcinoma, Adenosquamous carcinoma, Micropapillary carcinoma, Medullary carcinoma, Serrated carcinoma, Survival, Prognosis, Microsatellite instability, RAS mutation, BRAF mutation

## Abstract

Colorectal cancer is not just one type of cancer. Differences in outcome and reaction to treatment can at least be partly explained by different histological and molecular subtypes. Recognition of these differences may influence treatment decisions. However, there is huge variation in the amount of information that is available. Several tumour types such as mucinous carcinoma, signet ring cell carcinoma, neuroendocrine carcinoma and adenosquamous carcinoma have such a distinct phenotype that they are readily recognised. However, due to the rarity of signet ring cell carcinoma and adenosquamous carcinoma, limited data are available. More recently defined subtypes, like medullary carcinoma, serrated adenocarcinoma and micropapillary carcinoma, are not adequately diagnosed, which limits research possibilities using large-scale data from registries. In the current review, we systematically describe the histologic subtypes with the clinical and molecular background. We evaluate their prognosis compared to adenocarcinoma not otherwise specified and speculate about the clinical relevance.

## Introduction

Differences in oncological outcome of cancer have been long recognised, and with the apparently limitless possibilities of next-generation sequencing, it has been shown that at least part of these differences can be ascribed to the molecular background. However, the influence of tumour microenvironment should not be ignored and might very well be responsible for the limited responsiveness to therapy in a percentage of tumours [1]. Both molecular background and tumour-microenvironment interactions are closely associated with tumour type. This phenotypic determination of the character of tumour cells has been shown to be very relevant for prognosis. In colorectal cancer, mucinous carcinoma, the most frequent of the well-recognised subtypes, has a very limited response to systemic therapy in the metastatic setting, probably due to the distinct pattern along which the tumour disseminates [2]. Solitary liver metastases are uncommon in mucinous carcinomas, whereas peritoneal spread is more common.

**Fig. 1:**
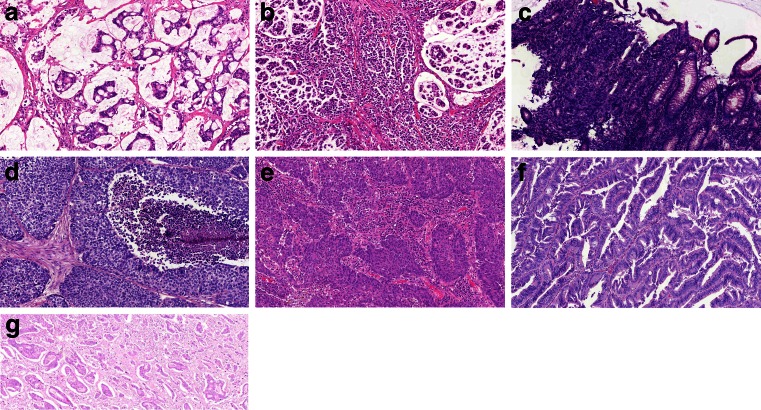
Different histological subtypes of colorectal cancer. **a** Mucinous carcinoma (MC), **b** signet ring cell carcinoma (SRCC), **c** neuroendocrine carcinoma (NEC), **d** adenosquamous carcinoma (ASC), **e** medullary carcinoma (MeC), **f** serrated carcinoma (SeC) and **g** micropapillary carcinoma (MiC)

Several subtypes have been recognised for a very long time, due to the clear differences in their phenotypes compared to the common adenocarcinoma (AC). Mucinous carcinoma (MC, Fig. 1a) is the most frequent phenotype in this group and therefore relatively well-studied. The other subtypes like signet ring cell carcinoma (SRCC, Fig. 1b), neuroendocrine carcinoma (NEC, Fig. 1c) and adenosquamous carcinoma (ASC, Fig. 1d) are very rare, and therefore, limited information can be gathered about their response to therapy. More recently, three additional subtypes have been defined, at least partly based on molecular and genetic studies. Medullary carcinoma (MeC, Fig. 1e) has a very strong correlation with microsatellite instability and Lynch syndrome, while serrated carcinoma (SeC, Fig. 1f) is strongly linked to the serrated pathway among which colorectal cancer can develop. Finally, in analogy with breast carcinoma and other tumour types, the micropapillary subtype has been recognised (MiC, Fig. 1g). In the general population, there is underreporting of these subtypes, because of insufficient recognition in daily practise. Evidence for aberrant clinical behaviour is only derived from single-centre studies and therefore needs validation in larger series.

In this review, we describe the different subtypes with the molecular background (if known) in correlation with clinical relevant issues and outcome data.

## Mucinous Carcinoma

In the spectrum of colorectal cancer (CRC), MC is the second largest histological subtype next to AC (10–15 % of CRCs) [1]. A tumour is designated as MC when more than 50 % of the tumour volume consists of extracellular mucus [3]. In the pools of mucus, malignant epithelium can be found in clumps of cells or as single cells [4]. The distinct histological presentation of MC has led to the hypothesis that these tumours may develop along a distinct oncogenic pathway, but exact mechanisms have not been elucidated to date. MCs have a tendency to be located in the right hemicolon (54–60 %), they present at a more advanced stage of disease, and high frequencies of MC are observed in Lynch syndrome patients (22–40 %) [1, 5–7]. The latter also explains the higher rate of microsatellite instability (MSI) that is seen in MC [7]. However, when MCs do develop according to the chromosomal instability pathway, they present with a markedly lower rate of chromosomal instability compared with ACs [8]. Other common molecular aberrations in MC are the higher rates of *KRAS*, *BRAF* and *PI3K* mutations, when compared with AC [7]. Constitutive activation of the RAS/RAF/MAPK and PI3K/AKT signalling pathways influences cell growth, survival, proliferation and cell motility, thus influencing tumour behaviour.

Since MC is diagnosed approximately once in every eight CRC patients, the prognostic value of this subtype has been studied extensively, but discussion remains. MC in general has long been considered an unfavourable prognostic indicator, but this has been disputed in various studies recently. In this perspective, the importance of the location of the primary tumour has been highlighted, since treatment strategies and prognosis vary for colon and rectal cancer patients.

For colonic MC, large population-based studies have demonstrated that there is no difference in overall survival after correction for stage at presentation [1, 5, 9]. Moreover, in a recent retrospective cohort study of 435 non-metastatic patients who were diagnosed with CRC between 2000 and 2010, it was demonstrated that MC was associated with an improved outcome (HR 0.75; 95 % CI 0.46–1.21). This favourable outcome has not been reported for rectal MC to date, and the controversy regarding prognosis in rectal MC patients has been ongoing over the past few decades. Rectal MCs tend to respond poorly to neoadjuvant therapies such as chemoradiotherapy, and high rates of incomplete resections have been reported [10, 11, 12••]. However, when adequate circumferential resection margins can be obtained during surgery, there is no difference in local recurrence rate and overall survival between rectal MC and AC [13]. Multidisciplinary assessment of rectal cancer patients including accurate imaging, optimal preoperative therapy and high-quality total mesorectal excision surgery has demonstrated to improve outcome for rectal MC [13] (Table 1).

**Table 1:**
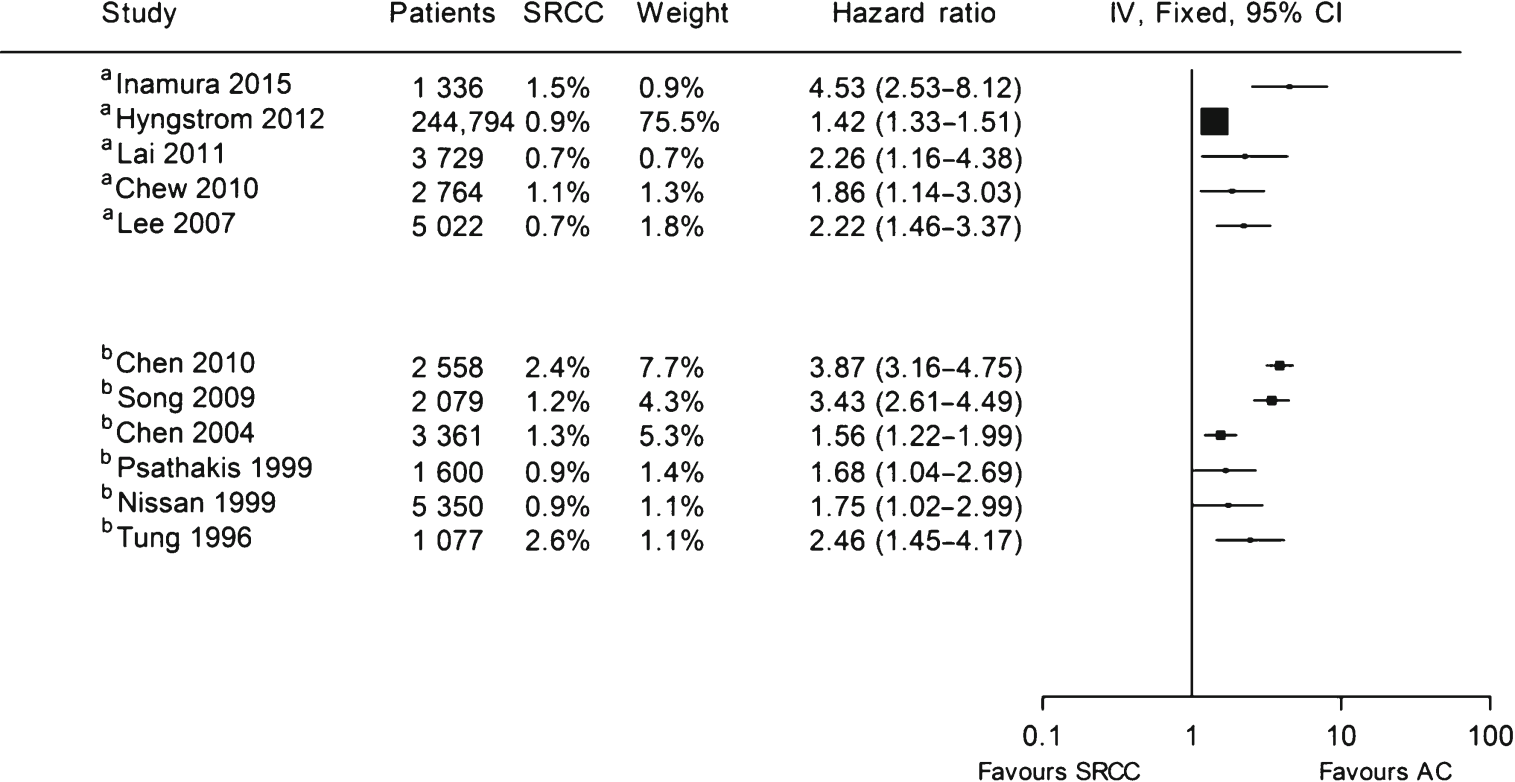
Overview of studies that analysed overall survival of colorectal SRCC patients compared with AC patients using multivariable or univariable analysis

Hazard ratios as reported in studies were used for analysis. If no hazard ratio was reported, it was calculated from the published data as described by Parmar et al. [69]

^a^Multivariable analysis

^b^Univariable analysis

As mentioned afore, MCs present at a higher stage of disease compared with ACs. Between 19 and 29 % of MC patients have metastatic disease upon their first presentation, and MCs are more likely to develop metastases during follow-up [1, 2, 5]. Although mechanisms are unclear, it has been demonstrated that MC has a distinct metastatic pattern of spread than AC. MC patients more frequently have metastatic disease in more than one organ. Compared with AC, hepatic metastases are less common in MC, and especially peritoneal metastases are frequently seen [2].

The deviant metastatic pattern of MCs is considered one of the possible explanations for the poor outcome after palliative chemotherapy as observed in various studies [12••, 14–17]. Median overall survival rates for MC patients from these studies are approximately 12 months, suggesting a reduced efficacy of chemotherapy for advanced-stage MC [12••]. In the adjuvant setting, no difference in outcome following chemotherapy has been observed when compared with AC [9, 12••, 18].

## Signet Ring Cell Carcinoma

Signet ring cell carcinoma (SRCC) is a rare subtype (1 % of CRCs) and is defined by the presence of the typical signet ring cells that comprise at least half of the tumour volume [1, 3]. Signet ring cells get their typical appearance as a result of a large mucus vacuole that displaces the nucleus to the edge of the cell. Compared with AC patients, SRCC patients are generally younger. Over 25 % of SRCC patients is under the age of 60 [1]. As for MC, SRCC is commonly found in the proximal (right) hemicolon in more than half of the patients [1, 19]. This corroborates very well with the high rate of MSI that is seen in SRCC tumours (24–40 %) [20, 21]. Especially SRCCs that also present with extensive mucus surrounding the tumour are likely MSI [20]. SRCCs are frequently of the CpG island methylator phenotype (48 %), and *BRAF* mutations are found in 30–33 % of SRCCs [20, 21]. *KRAS* is mutated in 53 % of SRCCs [21]. SRCCs are notorious for their rapid progression to an advanced stage of disease and are more likely to present with locally advanced and lymph node-positive tumours, with over three quarter of tumours being stage III or IV at the time of diagnosis [1, 5]. Perineural growth and lymphatic and vascular invasion are frequently present [20].

Approximately one third of SRCC patients already has metastatic disease upon the first presentation, and SRCC patients are more likely to develop metastases during follow-up [1, 2, 5]. SRCC has a distinct metastatic pattern, in which metastases develop at multiple sites (71 %). Liver metastases only account for 32 % of metastases in SRCC patients, and metastases to the peritoneum are seen in 51 %, compared with 73 and 20 %, respectively, in AC patients [2]. Especially peritoneal metastases in CRC are clinically challenging, but the introduction of cytoreductive surgery followed by hyperthermic intraperitoneal chemotherapy (HIPEC) has shown promising results and has improved prognosis for this patient group [22]. Unfortunately, for SRCC patients, a high rate of recurrence after HIPEC has been shown, resulting in a meagre median survival reaching only slightly over 1 year [23•, 24]. This had led to the recommendation to refrain from aggressive therapeutic approaches for peritoneal SRCC metastases in the presence of other poor prognostic factors [23•].

Prognosis for SRCC patients is not only poor for advanced-stage disease. SRCCs have stage-independent poorer outcomes when compared with AC [5, 25–33]. A study from 2015 showed that poorer outcomes were seen in both colon and rectal cancer patients separately, with 5-year relative survival rates of 30.8 and 19.5 %, respectively [1].

Despite the poor reported outcome, the clinical subset of SRCC patients is too small to being addressed in clinical studies, rendering insight into response to therapies limited. Although a poor outcome for metastatic patients following cytoreductive surgery and HIPEC has been described, benefit of adjuvant chemotherapy seems comparable with AC [1]. Additional analyses are highly needed and may give more direction in therapeutic strategies.

## Neuroendocrine Carcinoma

Neuroendocrine carcinomas form the end of a spectrum which ranges from adenocarcinomas with neuroendocrine differentiation via MANEC (mixed adenoneuroendocrine carcinoma) to NEC. With the introduction of immunochemistry and antibodies that were specific for neuroendocrine differentiation, large series of adenocarcinomas have been analysed. For the current review, we exclude the carcinoids, but in many studies, these are analysed in combination with NEC.

Differentiated neuroendocrine cells have been detected either scattered through the tumour or in distinct nests in approximately 20 % of adenocarcinomas [34]. When applying a definition of less than 30 % of neuroendocrine differentiation in tumour, a recent meta-analysis [34] including 1587 patients from 11 studies shows that neuroendocrine differentiation in adenocarcinomas is associated with a decreased 5-year survival rate (pooled OR 0.60, 95 % CI 0.37–0.97). This is in line with the recent publication of Shafqat et al. [35•].

MANEC was first defined in the 2010 edition of the WHO Blue Book [3] and describes tumours with both adenocarcinoma and neuroendocrine carcinoma components that each has a proportion of at least 30 %. Current literature is mainly limited to case reports. One study [36] compared 12 patients with MANEC with 27 patients with NEC and did not find any differences in survival.

There has been an increase in the incidence of colorectal neuroendocrine tumours over recent years [35•]; however, these tumours still account for a very small contingent of colorectal carcinomas, less than 1 %. From studies that compared the small adenocarcinoma components of these tumours with the neuroendocrine components [37–39], we know that both components have a clonal relationship, which can be traced back to the adenomatous stage. Mutational status (KRAS, BRAF, NRAS, PIK3CA, p53) in these tumours is not different from adenocarcinomas [39].

Compared to high-grade AC [35•], patients with NEC are younger, more often male. Tumours present as metastatic disease, and the primary location is more often in the rectum. In this large registry-based analysis, the 5-year overall survival of patients with NEC (*n* = 1367) was very poor, even compared with high-grade AC (*n* = 72,553), 16.3 versus 50.2 %. Similar data are obtained in an earlier SEER-derived study, where 455 NECs identified between 1992 and 2000 with a relative 5-year survival of 21.4 % were compared to all adenocarcinomas (5-year relative survival 62.1 %) [40]. This is in line with the data obtained from the NORDIC NEC study [41], where NECs derived from the colon and rectum demonstrate the worst survival rates of all NECs of the gastrointestinal tract.

## Adenosquamous Carcinoma

Like NEC, ASC is also part of a spectrum, which ranges from adenoacanthoma (i.e. adenocarcinomas with benign-appearing squamous metaplasia) through ASC to pure squamous carcinoma. The first and the latter are extremely rare. Cancer registries estimate the incidence of ASC between 0.06 and 0.09 % [42, 43]. Patient characteristics are not different from adenocarcinomas, but these tumours tend to present in advanced stages [42] and are located more often in the proximal and transverse colon, compared to AC. When they are located in the rectosigmoid area, the prognosis seems slightly better [43]. In early-stage disease (pT1-3N0), outcomes are comparable to AC; however, all other tumours have a significantly worse outcome, which is most pronounced in patients with synchronous metastases. Five-year overall survival is 23.5–25.4 % compared to 41.6–53.6 % in AC [42, 43]. However, these data are based on a total number of 244 patients. Molecular data and information about treatment response are not available.

## Medullary Carcinoma

MeC, previously known as solid-type poorly differentiated carcinoma and large cell minimally differentiated carcinoma, is characterised by sheets of malignant cells with vesicular nuclei, prominent nucleoli and abundant eosinophilic cytoplasm, exhibiting prominent infiltration by intraepithelial lymphocytes [3]. Single-centre studies estimate its frequency at approximately 4 % [44–46]. It is assumed that this type is underreported in cancer registries (0.08 %) [47], since it might be difficult to distinguish from poorly differentiated adenocarcinoma [48]. This subtype can be difficult to recognise, especially on preoperative biopsies, because of the undifferentiated appearance and the aberrant immunohistochemistry. Because of the lack of staining with cdx2 [49] and cytokeratin 20, it has been suggested that these tumours lose their intestinal differentiation. However, other intestinal markers, such as MUC1, MUC3 and TFF3, can still be demonstrated in MeC [50].

MeC is more frequent in female patients [45, 51] and has a very strong correlation with age, with an increased frequency in the elderly [51]. The preferred location is the right colon [44, 45]. These tumours are larger in size [44, 45], and upon diagnosis, it is hard to detect premalignant mucosa [44]. Compared to poorly differentiated adenocarcinoma, less nodal positivity and less extramural vascular invasion (EMVI) are present [44]. Compared to AC, more frequent lymphovascular invasion (LVI) is detected (62.9 versus 36.5 %) [45]. This subtype is invariably associated with MSI and a very high rate of BRAF mutations: 86 versus 69 % in other MSI tumours and 19 % or less in the general population [45].

In general, the prognosis is very good, compared to adenocarcinoma [44, 52]. The study of Knox et al. [45] failed to show this favourable prognosis in the univariate analysis, due to an unexplained high 30-day mortality in this group, but in the multivariate analysis there is an improved survival (HR 0.54, 95 % CI 0.30–0.96). There are no data about treatment response.

## Serrated Adenocarcinoma

The recognition of the serrated pathway as an important developmental route towards CRC has led to the proposition of SeC. This subtype is characterised by architectural similarity to sessile serrated lesions, with glandular serration, that may be accompanied by mucinous areas. The cells have a low nucleus-to-cytoplasm ratio [3]. However, all the available literature is from the same group of investigators, which limits the generalisability of the data. The data concerning SeC are lacking behind the enormous amount of information we are gathering about the serrated polyp [53].

Approximately 10 % of all CRCs can be classified as SeC [54]. One of the remarkable but also confusing features of SeC is that both a mucinous and a non-mucinous subtype exist. Actually, up to 45 % of all SeC occur in mucinous carcinomas [55]. In general, these tumours are more often proximally located [54] and have similar stage distribution when compared to AC. Microsatellite instability rates are not different from AC [55], but BRAF mutations are very frequent (33.3 versus 0 %) [56]. In addition, KRAS mutations are frequently observed (45.2 versus 27.1 %) [56]. These mutation rates are similar to the ones observed in serrated polyps. In the SeC, there is co-occurrence of KRAS mutations and MSI, which is absent in AC. When performing unsupervised clustering of expression data of SeC and AC, over 200 genes are differentially expressed [57], and almost perfect separation occurs. Large-scale microarray studies suggest that a molecularly defined subtype called CSS3 exists that might be exemplary for this subtype [58]. However, the proper histological classification of the tumours in this study has not been performed. A lack of response to anti-EGFR therapy independent of KRAS status has been suggested based on in vitro experiments [58]. The (unclassified) adenocarcinomas in this molecular subgroup also have a decreased prognosis; however, in the three available series of SeC, no difference in survival compared to AC has been observed [54, 57, 59].

## Micropapillary Carcinoma

This tumour is characterised by small clusters of tumour cells within stromal spaces mimicking vascular channels [3]. Initially described in breast cancer, the subtype has been recognised in other tumour types as well. The micropapillary component can be recognised in both AC as MC, and most studies define their tumour as MiC when they have at least a 5 % component.

The incidence of this component varies between 5 and 20 % of all colorectal carcinomas [60–62]. Data from cancer registries are not available. The age and gender of the patients are not different from AC, and location within the colon is also comparable [60–62]. Not all studies agree about the increased T stage which is sometimes described [60–62], but in all populations, the percentage of node-positive patients is high, up to 80 %, compared to an average of 40 % in the AC patients [60–64]. This is in accordance with almost double the incidence of LVI [60–62, 64]. EMVI is increased (46.7 versus 19.1 %), as is perineural invasion (PNI) [60, 63]. The risk of both positive nodes and LVI increases with an increasing percentage of the micropapillary component [60–62].

A number of studies have examined the molecular background of this new subtype. Microsatellite instability is not different from AC [61, 64] or possibly somewhat less frequent [60]. There is no difference in mutational spectrum (p53, KRAS, BRAF) [60]. The prognosis of this group of patients is worse [61, 63, 65]. The study of Lee [61] evaluated the effects of chemotherapy in 34 MiC patients, of which 27 were treated with systemic therapy, and in the survival curves, no difference can be observed. However, due to small numbers, no conclusions can be drawn. The decreased prognosis in combination with the sometimes observed high-grade cytologic features [60] has led to the suggestion that this tumour subtype should be graded as poorly differentiated [66] (Table 2).

**Table 2:** Summary of the different subtypes in comparison with adenocarcinoma n.o.s.

Type	Incidence (%)	Age	Gender	T	N	M	LVI	EMVI	MSI	RAS/RAF	Therapy	Survival
MC	10	=	Female	↑	↑	↑	?	?	↑	↑	=/↓	=
SRCC	1	↓	=	↑	↑	↑	↑	↑	↑	↑	=	Poor
NEC^a^	<1	↓	Male	?	↓	↑	?	?	=	=	?	Poor
ASC	<0.1	=	=	↑	↑	↑	?	?	?	?	?	Poor
MeC	4	↑	Female	↑	↓	↓	↑	↓	↑↑	BRAF	?	Good
SeC	10	=	Female	=	=	=	?	?	=	↑	aEGFR?	=
MiC	20	=	=	↑	↑	↑	↑	↑	=	=	?	Poor

*MC* mucinous carcinoma, *SRCC* signet ring cell carcinoma, *NEC* neuroendocrine carcinoma, *ASC* adenosquamous carcinoma, *MiC* micropapillary carcinoma, *MeC* medullary carcinoma, *SeC* serrated carcinoma, *LVI* lymphovascular invasion, *EMVI* extramural vascular invasion, *aEGFR* anti-EGFR therapy, *↑* higher, ↓ lower, = equal/similar, ? unknown

^a^NECs are compared with high-grade adenocarcinomas

## Conclusions

There is more than one type of CRCs. Increasing recognition of different developmental pathways important in both carcinogenesis and treatment response has led to a renewed interest in histological subtypes. While the molecular background of tumours is extremely important, the interaction with the microenvironment should not be forgotten. The presence of an extensive inflammatory infiltrate, as can be observed in MeC, is strongly associated with a very good prognosis [67•]. Indeed, we do observe a good outcome in this patient group, despite advanced T stage and increased presence of LVI. That there is more than the molecular background of tumours is also observed in MiC. Mutational status of these tumours seems comparable to AC; however, prognosis is significantly decreased, possibly due to the high frequency of LVI and EMVI, in combination with very high percentages of lymph node metastases.

Unfortunately, with the exception of MC, we have little data on response rates to chemotherapy in these subpopulations and hence very limited data about optimal treatment regimens. Careful histological and molecular analysis of tumours in clinical trials is required to fill this gap [68]. In addition, pathologists have to provide this information in the multidisciplinary meetings and explain the possible impact of these different subtypes properly. The issue of the different histologies of colorectal cancer should be put on the agenda of future oncology meetings to raise awareness on the different response rates, natural history and outcomes of the individual types.
